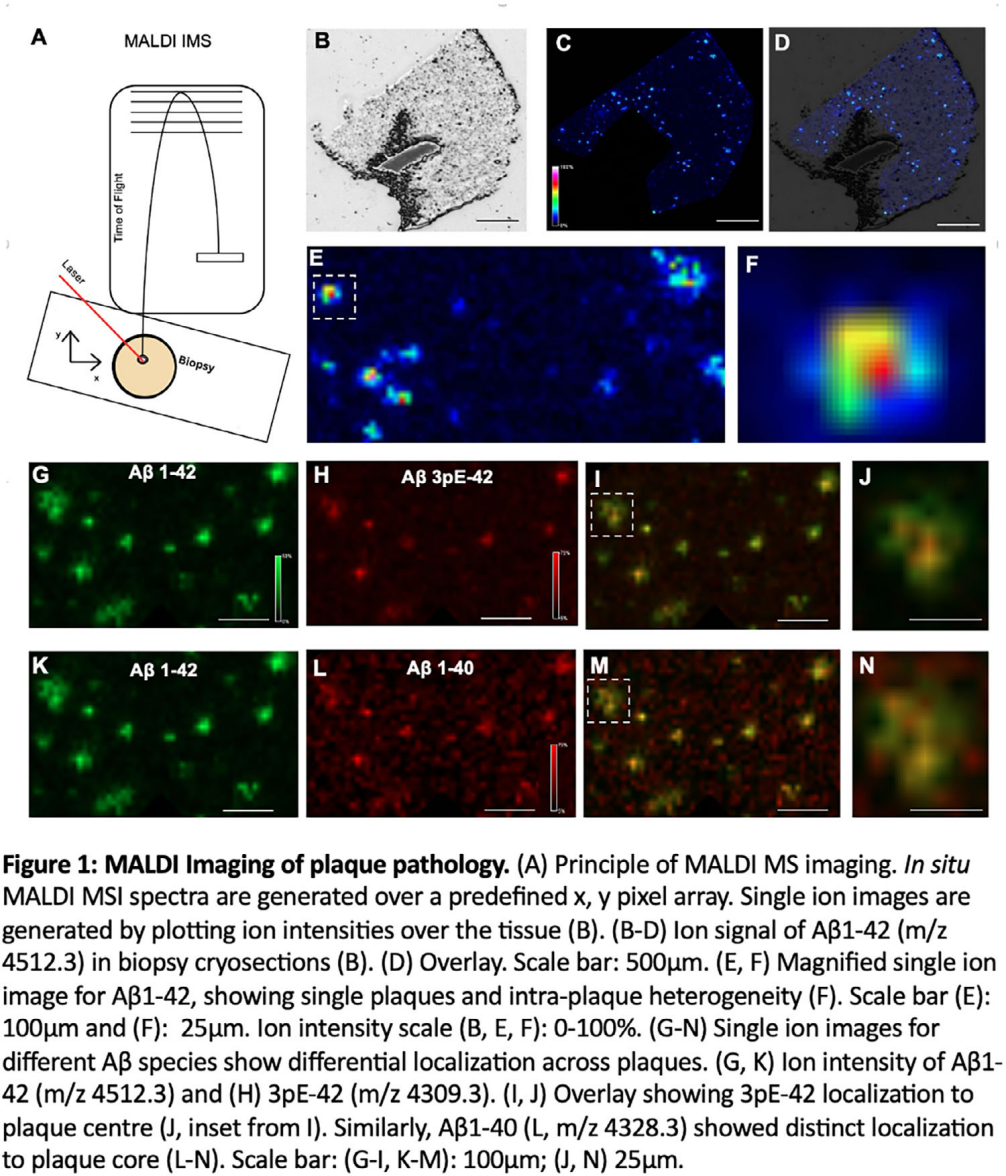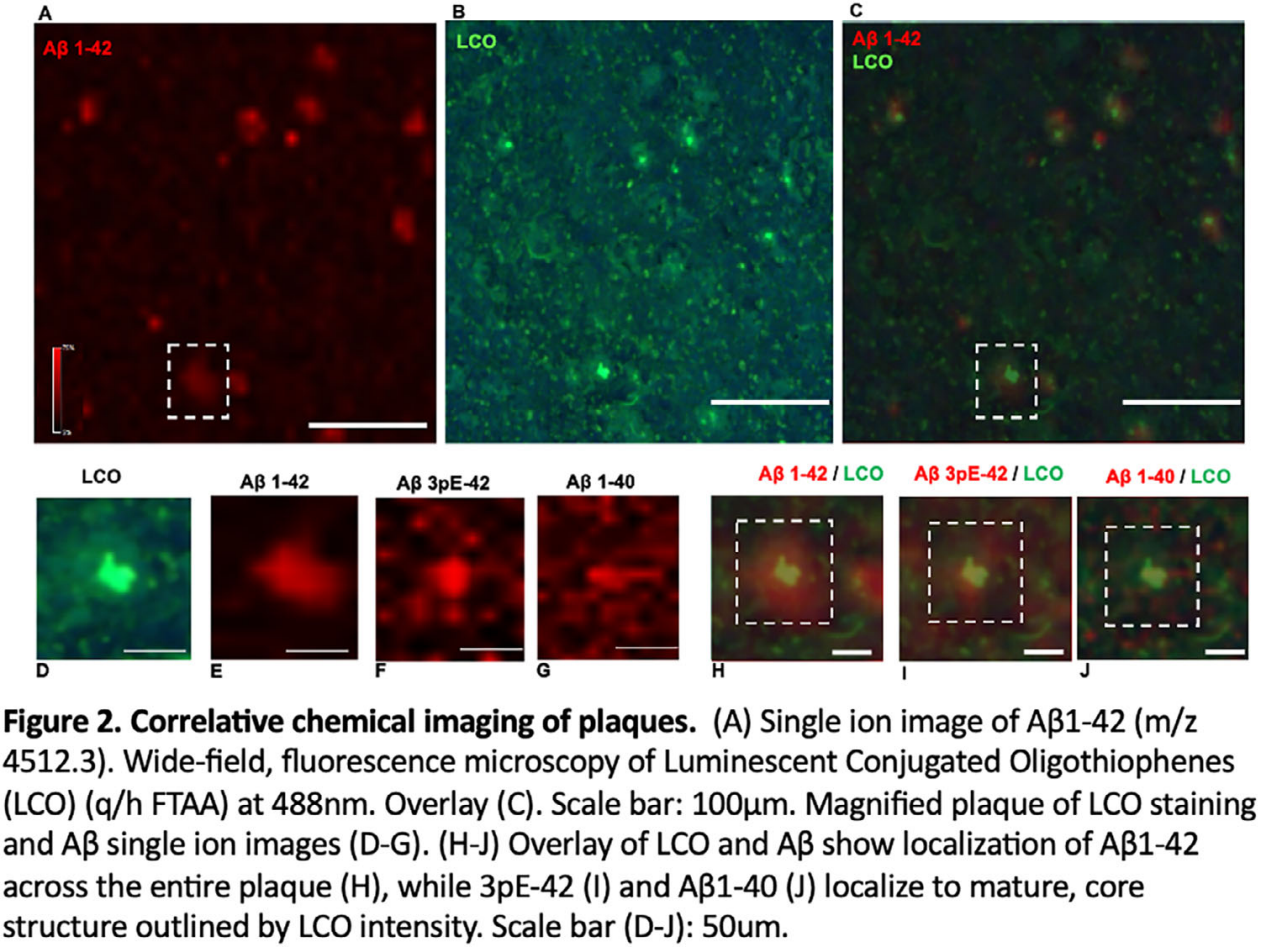# In vivo amyloid and tau metabolism in human brain tissue

**DOI:** 10.1002/alz.091279

**Published:** 2025-01-03

**Authors:** Aram Aslanyan, Soumya Mukherjee, Maciej Dulewicz, Reid Coyle, Yingxin He, Chihiro Sato, Nupur Ghoshal, Junyue Ge, Eleanor Moncur, Srinivas Koutarapu, Elena Camporesi, Lewis Thorne, Ahmed Toma, Katherine Schwetye, Kaleigh F Roberts, Laurence Watkins, Donald L. Elbert, Randall J. Bateman, Jörg Hanrieder, Ross W. Paterson

**Affiliations:** ^1^ Dementia Research Centre, UCL Queen Square Institute of Neurology, London United Kingdom; ^2^ Washington University School of Medicine, St. Louis, MO USA; ^3^ Institute of Neuroscience and Physiology, Sahlgrenska Academy at the University of Gothenburg, Gothenburg Sweden; ^4^ National Hospital for Neurology and Neurosurgery, London United Kingdom; ^5^ University of Washington, Seattle, WA USA; ^6^ Department of Neurodegenerative Disease, UCL Queen Square Institute of Neurology, University College London, London United Kingdom; ^7^ Darent Valley Hospital, Dartford United Kingdom

## Abstract

**Background:**

Knowledge of the chemical composition of amyloid plaques and tau tangles at the earlier stages of Alzheimer’s disease (AD) pathology is sparse. This is due to limited access to human brain during life and at the earlier stages of AD pathophysiology and technical limitations in quantifying amyloid and tau species at a subcellular level. Understanding the chemical composition of plaques and tangles, how rapidly they grow and what factors drive growth is important for developing and refining therapeutics. We access *in vivo* cortical brain biopsy samples from individuals undergoing surgery, aiming to provide detailed characterisation of pathology with spatial and temporal resolution.

**Method:**

We collected *in vivo* brain biopsies with matched ventricular and lumbar cerebrospinal fluid samples from individuals with suspected Normal Pressure Hydrocephalus (NPH) undergoing ventriculoperitoneal shunt surgery. All participants were labelled with intravenous ^13^C_6_ Leucine as per Stable Isotope Labelling Kinetics protocol. We used immunohistochemistry, immunoprecipitation‐mass spectrometry (IP‐MS) and Matrix Assisted Laser Desorption Ionization (MALDI) mass spectrometry‐based imaging (MSI) to characterise amyloid and tau and their common post‐translational modifications. We calculate the tracer‐to‐tracee ratio of labelled to unlabelled amyloid and tau to determine rate of pathological accumulation of these proteins. Using a 2‐compartment model we establish the half‐life of tau in brain tissue.

**Result:**

We collected cortical brain biopsies from individuals with suspected NPH (n = 6), labelled between 4.25 hours to 133 days prior to surgery; 4 out of 6 individuals had amyloid plaques (range 1‐25 plaques/mm2). These individuals had mild cognitive impairment. Cored plaques showed characteristic localization of Aβ1‐40 and Aβ 3pE‐40 in the core while x‐42 species including 1‐42, 4‐42 and 3pE‐42 showed a more homogenous distribution across both plaque core and diffuse/immature plaque periphery (Figure 1, 2). We did not detect incorporation of labelled amyloid in any subject using IP‐MS or MALDI. We detected labelled tau in brain homogenate as early as 4.25 hours after label administration. The half‐life of tau in human brain was calculated to be 26.85 days.

**Conclusion:**

Our study provides the first detailed chemical characterisation of AD pathology in living human brain giving insights into plaque composition, age and tau dynamics.